# Sudden Cardiac Death and Channelopathies: What Lies behind the Clinical Significance of Rare Splice-Site Alterations in the Genes Involved?

**DOI:** 10.3390/genes15101272

**Published:** 2024-09-27

**Authors:** Mauro Pesaresi, Alessia Bernini Di Michele, Filomena Melchionda, Valerio Onofri, Federica Alessandrini, Chiara Turchi

**Affiliations:** 1 Section of Legal Medicine, Department of Biomedical Sciences and Public Health, Polytechnic University of Marche, Via Tronto, 60126 Ancona, Italy; m.pesaresi@univpm.it (M.P.); a.bernini@pm.univpm.it (A.B.D.M.); f.melchionda@univpm.it (F.M.); f.alessandrini@univpm.it (F.A.); 2 Legal Medicine Unit, AOU Azienda Ospedaliero Universitaria delle Marche, 60126 Ancona, Italy; valerio.onofri@ospedaliriuniti.marche.it

**Keywords:** sudden cardiac death (SCD), channelopathies, molecular autopsy, splice-site variant, variant of unknown significance (VUS)

## Abstract

**Background and objectives**: Sudden cardiac death (SCD) is a natural and unexpected death of cardiac origin that occurs within 1 h from the onset of acute symptoms. The major leading causes of SCD are cardiomyopathies and channelopathies. In this review, we focus on channelopathies, inherited diseases caused by mutations affecting genes encoding membrane ion channels (sodium, potassium or calcium channels) or cellular structures that affect Ca^2+^ availability. The diagnosis of diseases such as long QT, Brugada syndrome, short QT and catecholaminergic polymorphic ventricular tachycardia (CPVT) is still challenging. Currently, genetic testing and next-generation sequencing allow us to identify many rare alterations. However, some non-coding variants, e.g., splice-site variants, are usually difficult to interpret and to classify. **Methods**: In our review, we searched for splice-site variants of genes involved in channelopathies, focusing on variants of unknown significance (VUSs) registered on ClinVar up to now. **Results**: The research led to a high number of splice-site VUSs of genes involved in channelopathies, suggesting the performance of deeper studies. **Conclusions**: In order to interpret the correlation between variants and pathologies, we discuss experimental studies, such as RNA sequencing and functional analysis of proteins. Unfortunately, as these in vitro analyses cannot always be performed, we draw attention to in silico studies as future perspectives in genetics. This review has the aim of discussing the potential methods of detection and interpretation of VUSs, bringing out the need for a future reclassification of variants with currently unknown significance.

## 1. Introduction

Sudden death (SD) is described as a natural and unexpected death that occurs within 1 h from the onset of acute symptoms. It affects apparently healthy people and subjects whose disease is not sufficiently severe to prognosticate a fatal outcome [1]. Sudden cardiac death (SCD) is the cause of about 85% of all sudden deaths. Western countries have a high incidence of SCD, making it a leading cause of death. Studies conducted in the United States, Europe and China estimated that SCD has rates in the general population that range from 50 to 100 per 100,000 people per year [2]. Moreover, aging leads to an increase in the annual incidence of SCD; particularly, it has been shown that SCD occurs most frequently between birth and 6 months of age (sudden infant death syndrome—SIDS), and its incidence is 100-fold lower in individuals under 35 years old (0.001%) than in those over 35 years of age [3,4,5,6,7]. However, the incidence is higher in men than in women at any age [2]. 

Generally, when sudden death affects young people (under 35 years), SCD is more likely to be the result of a heart attack caused by electrical malfunction. Mostly, SCD is provoked by inherited cardiomyopathies or congenital cardiac channelopathies [8]. Cardiomyopathies are mainly associated with cardiac structural abnormalities that lead to arrhythmias. In some peculiar cases, the causes of cardiomyopathy are heterogeneous and not easily diagnosed, since they may depend on genetic alterations that are not reflected in the structure of the heart. Alterations may affect structural proteins, such as those of sarcomeres, desmosomes and the cytoskeleton [9,10,11]. In both young people and adults, the most common SCD-related cardiomyopathies are hypertrophic cardiomyopathy (HCM), dilated cardiomyopathy (DCM), arrhythmogenic right ventricular cardiomyopathy (ARVC), restrictive cardiomyopathy (RCM) and left ventricular non-compaction (LVNC) [11,12].

Cardiac channelopathies are disorders of ion channels that predispose individuals to alteration of the normal heart rhythm. They may lead to tachyarrhythmia (heartbeats that are too rapid) or to bradyarrhythmia (heartbeats that are too slow). Both cases may lead to circulatory collapse and, in severe cases, sudden death. Most channelopathies are genetically inherited, although some can also be acquired and secondary to drugs, toxins or autoimmune diseases. Genes encoding membrane ion channels (sodium, potassium or calcium channels) or proteins that regulate them can be affected by pathogenic mutations, leading to congenital cardiac channelopathies [13,14]. The main cardiac channelopathies include long-QT syndrome (LQTS), short-QT syndrome (SQTS), Brugada syndrome (BrS) and catecholaminergic polymorphic ventricular tachycardia (CPVT) [13,15]. With few exceptions, these disorders are inherited in an autosomal dominant pattern with incomplete penetrance and variable expressivity [15]. For these reasons, it is difficult to give a diagnosis based solely on a physical examination. Genetic testing could support a diagnosis by identifying the disease-causing mutation. However, up to 20% of patients with LQTS and BrS, 80% with SQTS and 40% with CPVT may receive negative results from genetic testing [16].

In this review, we want to highlight that there is still a lot to learn about the genetics of channelopathies, especially less studied rare variants within non-coding regions, such as splice-site variants. A splice-site variant is a genetic alteration in the DNA sequence (a deletion, an insertion or a change in a nucleotide) that occurs in the region between an exon and an intron (a splice site). Often, splice-site variants are classified as pathogenic, likely pathogenic or a variant of unknown significance, following the American College of Medical Genetics and Genomics (ACMG) guidelines. Indeed, these alterations can lead to the retention of large segments of intronic DNA, or they may lead to the splicing out of entire exons, resulting in the production of a different mRNA molecule translated into a non-functional, truncated protein, causing defects in ion channels and consequently channelopathy.

Taking into account the lack of diagnosis and the non-conclusive genetic testing of the DNA of patients suffering from cardiac channelopathies, we want to focus the discussion on transcriptome analysis (mRNA studies) to understand the molecular significance of splice-site variants in mRNA molecules.

## 2. Overview of the Most Common Channelopathies

Channelopathies are pathologies caused by channel dysfunctions that affect the electrical functioning of the heart, predisposing it to bradyarrhythmias or tachyarrhythmias in the absence of a structural heart disorder [17,18]. Cardiac ion channels are macromolecular complexes assembled at specific sites within the membranes of cardiomyocytes [19]. Ion channels are characterized by two features: ion permeation and gating. Ion permeation is the movement through an open channel of specific ions (Na^+^, K^+^ and Ca^2+^), while gating describes the opening and closing mechanisms of ion channels. The gating mechanisms of ion channels are divided into voltage-dependent, ligand-dependent and mechanosensitive subclasses [20]. The opening and closing of ion channels expressed on the sarcolemmas of cardiomyocytes results in the generation of an action potential (AP). The various phases of the cardiac action potential are associated with changes in cell membrane permeability, mainly to Na^+^, K^+^ and Ca^2+^. By gating, ion channels allow ion currents across the sarcolemma, thereby creating the five phases of AP. Phase 0 is characterized by a rapid depolarization, during which the membrane potential shifts to a positive voltage range (+50 mV). In ventricular cells, this is a consequence of the activation of sodium channels and the inflow of sodium ions into the cells. In contrast, in sinoatrial node cells, the increase in membrane voltage is mainly caused by the influx of calcium ions due to the activation of L-type calcium channels. Phase 1 is characterized by a rapid inactivation of sodium channels and the consequent reduction in the flux of sodium ions into the cell. Phase 2 or the plateau phase is characterized by a constant membrane potential due to the balance of ions moving into and out of the cell; potassium ions move out of the cell and calcium ions move into the cell. This phase determines the long duration of the AP, and it has an important role in preventing cardiac arrhythmia. Phase 3 involves a rapid repolarization of AP due to the closing of calcium channels, while potassium channels remain open and potassium ions flow out of the cell until the membrane potential is restored to about −90 mV (phase 4). The resting potential phase (phase 4) is stable at about −90 mV in ventricular cardiomyocytes; it results from the balance of the flux of sodium and calcium ions into the cell and the flux of potassium, chloride and bicarbonate ions out of the cell [21]. In physiological conditions, as described above, normal cardiac excitability results from a balance of depolarizing and repolarizing ionic currents. In pathological conditions, abnormal, inhomogeneous repolarization and alteration of the depolarization process underline ventricular tachycardia or ventricular fibrillation—characteristic arrhythmias of many cardiac channelopathies [22].

Major cardiac channelopathies include long-QT syndrome (LQTS), short-QT syndrome (SQTS), Brugada syndrome (BrS) and catecholaminergic polymorphic ventricular tachycardia (CPVT) [10,13,14].

Long-QT syndrome (LQTS) was the first genetically identified channelopathy, and it is the most common form of congenital cardiac disease involving channels. As the name suggests, it is defined by prolonged ventricular repolarization (long QT intervals), which predisposes to a high risk of ventricular tachyarrhythmias (e.g., torsade de pointes), syncope and sudden cardiac death [17]. To date, 16 types of LQTS have been classified (Supplementary Materials: Table S1) [23], each of them caused by alterations in genes that encode ion channels or related proteins [24]. Acquired factors can also contribute to the occurrence of LQTS, such as acquired diseases, drugs and electrolyte abnormalities (hypocalcemia, hypokalemia and hypomagnesemia) [17].

Among patients with LQTS who undergo genetic testing, 90% of LQTS cases are positively genotyped as LQT1 or LQT2, while LQT3 probably accounts for about 5% to 8% of cases and the remaining types of LQTS are extremely rare [25].

LQT1, the most common form of LQTS, is caused by loss-of-function mutations in the *KCNQ1* gene, which encodes the α-subunit of the Kv7.1 potassium channel. The delayed rectifying potassium current that is dominant during the phase of repolarization is the result of the malfunctioning of this tetrameric protein. Alterations to the gene encoding the Kv7.1 potassium channel reduce the repolarizing currents required to terminate the cardiac action potential, leading to a prolongation of the QT interval [26].

LQT2 is due to mutations in the *KCNH2* gene on chromosome 7. *KCNH2* encodes HERG, the α-subunit of the delayed rapid rectifier potassium channel responsible for conducting the slow rectifier current, which is responsible for determining how long the plateau phase and repolarization phase of ventricular cardiomyocyte repolarization last. In these patients, auditory stimuli are the main cause of arrhythmias [27].

LQT3 is the third most common form of LQTS and is caused by a gain-of-function mutation of the *SCN5A* (sodium channel protein type 5 subunit α) gene on chromosome 3, which encodes the α-subunit of the cardiac voltage-gated sodium channel Nav1.5. This counterbalances the repolarization process mainly through an alteration of the channel inactivation process, leading to a late/persistent sodium current at the end of the cardiac action potential.

Brugada syndrome (BrS) is a cardiac channelopathy inherited in an autosomal dominant pattern. BrS predisposes patients to fatal cardiac arrhythmias, and it accounts for 20% of sudden cardiac deaths that occur in the absence of gross structural cardiac abnormality [28,29]. The prevalence of BrS is approximately 3 to 5 per 10,000 people, and it is 8 to 10 times more common in males than females [30]. The mutation most frequently observed in BrS (found in 15–30% of cases) is a loss-of-function mutation in the gene *SCN5A* (BrS1) [31,32,33], which encodes a cardiac voltage-gated sodium channel subunit.

Other types of Brugada syndrome are due to mutations in genes encoding proteins that interact with ion channels, such as GPD1L (BrS2); genes encoding ion channels that carry calcium or potassium ions, such as *CACNA1C*, *CACNB2* and *KCNE3* (BrS3, BrS4 and BrS5, respectively); and genes responsible for proteins which form sodium channel β subunits (*SCN1B*). See Table S2 in the Supplementary Materials [34,35].

Short-QT syndrome (SQTS) is a rare, sporadic or autosomal dominant channelopathy characterized by a constantly short QT interval and accelerated cardiac repolarization manifested by atrial fibrillation, syncopal episodes and/or sudden cardiac death in patients with no underlying structural heart disease [36]. To date, it has been found that gain-of-function mutations in genes encoding potassium channels (*KCNQ1*, *KCNH2* and *KCNJ2*) and loss-of-function mutations in genes encoding calcium channels (*CACNA1C* and *CACNA2D1*) result in an abbreviated repolarization phase during action potentials and shortening of the QT interval. The classified types of SQTS are listed in Table S3, Supplementary Materials [37]. Nevertheless, SQTS has yet to be studied in depth, since about 40% of patients do not have an identified genetic cause.

The last type of channelopathy we discuss is catecholaminergic polymorphic ventricular tachycardia (CPVT). This inherited cardiac condition is characterized by arrhythmias that occur during physical stress or acute emotion, which can induce dizziness, syncope and/or sudden cardiac death. The most common CPVT is the autosomal dominant form caused by mutations in the *RYR2* gene (CPVT 1), which encodes the cardiac ryanodine receptor 2 (RYR2) [38]. RYR2 channels are responsible for releasing calcium from the sarcoplasmic reticulum into the cytosol when the cell membrane is depolarized. The result of defective closure of RYR2 channels is intracellular calcium leakage, which increases the potential for delayed after-depolarizations and subsequent ventricular tachycardia [39]. The second genetic variant of the disease (CPVT 2) is responsible for less than 5% of cases, and it is associated with an autosomal recessive mutation of the *CASQ2* gene, encoding cardiac calsequestrin. Calsequestrin is a protein that plays a vital role in regulating the storage and release of Ca^2+^, and, through its interaction with other proteins, it acts as a significant regulator of RYR2 channels [40,41]. The other types of CPVT are listed in Table S4 in the Supplementary Materials, but they have rarely been observed [42].

## 3. Mutations in Genes Involved in Channelopathies: Focus on Splice-Site Variants

As for other diseases, different types of point mutations can affect the genes involved in channelopathies: deletions, insertions and substitution of bases in DNA sequences. A mutation can affect a coding sequence or a non-coding sequence.

When a mutation affects a coding sequence, it could lead to a frameshift mutation if caused by the insertion or deletion of a number of nucleotides that are not evenly divisible by three, or it could lead to a point mutation due to a substitution, which means a change in a single nucleotide. A point substitution mutation can be synonymous or non-synonymous. A synonymous substitution consists of the replacement of a codon with another codon that codes for the same amino acid, creating an amino acid sequence like the original one (silent mutation). By contrast, a non-synonymous substitution substitutes a codon with another codon that does not code for the same amino acid. This is called a missense mutation when the genetic variant produces an amino acid which is different from the usual one at that position. Some missense variants will change the function of a protein. If a point mutation results in a premature stop codon, it is called a nonsense mutation, and it results in a truncated and often non-functional protein product.

There are many mutations in coding regions of genes that encode ion channels which lead to the onset of diseases, and they have been studied and well described in the literature.

Less frequently, we have heard about non-coding DNA, which used to be called junk DNA because these regions do not code for proteins. Fortunately, this definition is no longer used today, as important functions of non-coding regions have been discovered and their role should not be underestimated in many pathologies.

The non-coding regions of DNA fractions include regulatory sequences that control gene expression, scaffold attachment regions, origins of DNA replication, centromeres and telomeres, introns, pseudogenes, intergenic DNA and some non-coding RNA molecules, such as miRNAs, tRNA, rRNA, piRNA and regulatory RNA. Alterations could occur at splice sites too. A splice site is a sequence region within a non-coding site of an intron directly next to a coding sequence of an exon. Splice-donor and splice-acceptor sequences are responsible for controlling the splicing process, where a ribonucleoprotein called a spliceosome cuts the sequence. Alterations in these sequences may result in retaining large portions of intronic DNA or in the splicing out of entire exons, potentially resulting in the production of a non-functional protein [43].

Reported results in the literature show that the most common genes involved in channelopathies have rare alterations in splice sites.

Leong et al. [44] illustrated that only 20% of all splice variants reported in the *KCNQ1*, *KCNH2* and *SCN5A* gene entries in the HGMDPro 2015.4 database have been evaluated using transcriptional assays. So, further work may be required at the protein level to determine the effect that splice variants have on protein function.

Regarding Brugada syndrome, disease-causing mutations in SCN5A coding regions are observed in approximately 20% of cases; other genes have been associated with the disease, but their role is disputed [45,46]. In general, despite several genes having been reported as potentially associated with BrS, LQTS, SQTS and CPVT, only a few of them are considered definitively causative [46]. Moreover, as new genetic studies appear, it is becoming more evident that both coding and non-coding regions play a fundamental role in the pathophysiology of inherited human diseases. However, the effect of variants in non-coding regions is difficult to assess.

## 4. Methods of Detection and Analysis of Splice-Site Alterations

When a geneticist performs a routine genomic DNA analysis on a patient suffering from a suspected genetic disease such as a channelopathy, the first step is DNA sequencing. The DNA analysis is usually performed starting from fresh whole blood in EDTA or frozen blood stored at −20 °C, with the analysis performed later [47].

Only after DNA sequencing do we become aware of the presence of a variant by identifying its type and position. When a rare or novel variant in a genomic sequencing sample is detected, the American College of Medical Genetics and Genomics (ACMG) guidelines [48] recommend evaluating not only the significance of the variant but also the context of the patient’s and family’s history. This modus operandi is described in a generic way for any variant at any position in the genome. It is highly suggested to include so-called “trio” testing (mother, father and affected child) in the setting of whole-genome sequencing. Testing other family members is important to establish when a variant is de novo, when a variant co-segregates with disease in the family and when a variant is in trans with a pathogenic variant in the same recessive disease-causing gene. Family segregation tests make interpretations much more efficient and conclusive.

Moreover, when a non-coding variant appears, it could be useful to perform other experimental studies, such as RNA studies through next-generation sequencing (NGS), to understand its clinical significance. The encountered problems are related to the availability and the condition of the biological starting material and its storage. Usually, blood samples in EDTA are stored at +4 °C for 1–2 weeks or −20 °C for longer periods, with the aim of extracting DNA. However, when blood samples are stored at +4 °C, the different biochemical processes, although less efficient, still work, leading to apoptosis and internal RNA degradation, which could affect the gene expression profiles [46]. Specifically, in disease-related studies, these changed genes could make the output distorted and confusing [38]. Whole blood may be stored at −20 °C, which is suitable for DNA analysis but not for further studies on RNA. In fact, at −20 °C, ribonucleases are still active and RNA degradation is not prevented [49].

We run into another issue when we need to perform more than one analysis and we repeatedly freeze and thaw the same sample. Repeated freeze/thaw cycles invariably damage the plasma membrane, causing the discharge of RNase from ruptured cells into the plasma and consequent RNA degradation. So, it could be appropriate to store whole blood in aliquots, one for the DNA analysis (which could be stored at +4 °C or −20 °C) and at least another for RNA analysis (stored at −80 °C) [50,51]. In the case of RNA analysis, it is also suggested to thaw frozen samples quickly in a water bath at 37 °C [52,53,54,55,56,57].

Moreover, as shown by Yamagata et al. [46], another point needs to be discussed: the stabilization of RNA. This could be achieved using RNA-stabilizing reagents, but they are expensive and not always available in general clinics. In this regard, we would like to mention the guidelines for autopsy investigation of sudden cardiac death [58], which describe the storage modalities of each biological sample for further laboratory genetic tests. In addition, we would like to highlight the importance of a standardized protocol or updated reporting guidelines for sample storage for functional RNA analysis. In fact, DNA sequencing simply gives us an informative result about the type of variant and its location; it provides no knowledge of its clinical significance. Only with a functional study or by consulting updated databases of in silico tools can we trace its classification as a benign/likely benign/likely pathogenic/pathogenic/VUS variant. Considering an alteration in a non-coding sequence, it is not always easy to reconstruct the amino acid sequence and understand the changes in the protein with a functional study.

In the ClinVar public archive (https://www.ncbi.nlm.nih.gov/clinvar/, accessed on 10 July 2024), we looked for the genes most involved in channelopathies by gathering the number of total mutations with a pathogenic/likely pathogenic/VUS (variant of unknown significance) clinical relevance. We then selected the splice-site variants as “molecular consequences” and observed that VUSs are more frequent than pathogenic/likely pathogenic variants. The collected data are shown in Table S5 in the Supplementary Materials, and we can see that more than 60% of splice-site variants are VUSs. Moreover, it is estimated that 15% of genotype-elusive disease cases are due to altered splicing resulting from intronic variations [58,59], suggesting that splice-site variants are not well studied yet.

The ACMG guidelines [58] recommend that functional analysis using either RNA or protein analysis is essential to confirm the impact of splice-site variants, even though they are predicted to lead to a null effect [60]. A splicing variant could maintain the critical domains of the protein and thus lead to a mild or neutral effect with a smaller length change or a function-gain effect.

Otherwise, splice-site variants in position +/−1 or 2 are classified as PVS1 (very strong evidence of pathogenicity), since they often disrupt the gene’s function, resulting in the absence of the gene product due to lack of transcription or in an altered transcript. The critical domains of the protein could be retained by an in-frame deletion or insertion caused by splice-site variants, resulting in a mild or neutral effect that has a minor length change or a gain-of-function effect.

Functional studies can be a powerful tool for supporting pathogenicity, but not all of them can predict an impact on a gene or protein function [58], and they are not always easy to carry out. Assuming this, it could be informative to perform in silico studies, which have recently gained interest in genetics. In this regard, ACMG guidelines are provided for studies that aim to predict splice sites. The in silico predictive tools are computational studies. They include multiple software programs developed to predict splicing. Splice-site prediction tools have generally improved their sensitivity (~90–100%) and specificity (~60–80%) in predicting splice-site abnormalities [61]. There are many updated articles and reviews about splicing prediction tools in silico, comparing one with another [62,63]. Having many tools allows us to strengthen predictions and add information about splicing sites and the alterations themselves. On the other hand, the large amount of in silico prediction tools may be a problem regarding the choice and interpretation of results. Numerous of them employ different algorithms, which are actually based on similar assumptions. Analyses cannot be strengthened by combining predictions from different tools, and these should be considered as a single source of evidence in variant interpretation [58]. Usually, when all in silico analyses lead to the same prediction, this evidence can be considered as supporting a classification. If the in silico predictions do not match, this evidence should not be used to classify a variant. Furthermore, it should be noted that the availability of experimentally validated variants depends on the continuous upgrading of variant databases by users. For these reasons, it is not recommended to use in silico tools as the only source of evidence to make a clinical assertion. It is worth characterizing splice variants based on in vitro assays, such as RNA analysis or proteomic tests [64].

As described by the ACMG guidelines, when a computational test is performed and the splicing variant suggests a possible impact on the disease, the variant is classified as a VUS until an in vitro test is performed to exclude or confirm the pathogenic role [58]. This could explain the high number of VUSs in genes involved in channelopathies, as functional tests are often not performed (Table S5, Supplementary Materials). So, generally, when a genetic test is performed on a DNA sequence in a laboratory and a splice-site variant is found, the first step of analysis could be an in silico approach, using tools that are able to predict the effect of the variant. This primary approach could be conclusive if the molecular effects of the variant found are well known. But, usually, an in vitro test is always necessary to assess the role of a variant. Validation methods can be performed on RNA sequences, mainly through reverse transcriptase PCR (RT-PCR) and RNA sequencing and/or at the protein level through functional studies such as protein truncation tests (PTTs).

As mentioned above, although functional analysis is routine in genetic laboratories, RNA manipulation is not so easy due to the degradation rate of the molecule. Also, the choice of the biological starting material as the optimal source for RNA analysis is not to be underestimated. Blood represents the best choice for isolating a huge amount of RNA from patients to detect splicing variants [65,66]. On the other hand, tissues may be the best biological matrices to compare effects resulting from aberrant splicing in both healthy and affected samples, and such comparisons should definitively establish if a splicing alteration causes disease. The limitation to consider is that the appropriate tissue is not always available, and, if available, it is usually fixed in formalin and embedded in paraffin (FFPE tissue), so it is difficult to reach a high yield of RNA [66,67]. Proteomic tests are based on protein function studies and, different from functional studies on RNA molecules, they are usually performed by immunochemistry. In this context, the protein extraction procedure has limitations that could lead to contamination. The tests most commonly performed are the PTT or in vitro synthesized protein assay (IVSP) [68] developed to identify variants that contain a premature stop codon, which can compromise protein translation. These methods are simple and fast, and they screen for biologically relevant gene variants. A very interesting study performed by O’Neil et al. [69] used molecular engineering techniques to try and identify the biological consequences of a splice-site variant and, possibly, reclassify variants currently known as VUSs. The researchers implemented two functional assays: the minigene assay and CRISPR genome editing. Minigenes were constructed to test RNA effects as cis-regulatory elements and the gene expression associated with splicing factors and proteins as trans-regulatory elements.

The minigene assay was used to understand the role of the splice-site variants in the diseases. They compared a WT (wild-type) minigene construct with the mutated one through RT-PCR product gel band size and confirmed the result by Sanger sequencing.

Since this technique was found to be suitable only for canonical AG-GT splice sites, O’Neil et al. performed another in vitro study by introducing the variant with CRISPR-Cas9 into healthy control iPSC-CMs (induced pluripotent stem cell-derived cardiomyocytes), assessing splicing consequences by RT-PCR of isolated RNA. The results obtained from both in vitro assays were compared to a computational test called SpliceAI, demonstrating that the experimental findings were concordant with in silico results. The functional and computational findings were then integrated with the ACMG criteria [70] to reclassify the variants of unknown significance, allowing a better interpretation of their role in the pathogenesis.

## 5. Conclusions

In rare hereditary diseases, e.g., channelopathies, a proper classification of non-coding sequences, such as splice variants, is crucial for an accurate diagnosis. In patients with suspected channelopathy, after DNA sequencing, we can repeat blood sampling to perform other in vitro tests, such as RNA sequencing or functional tests.

In conclusion, having collected in Table S5 a considerable number of variants of unknown significance in the splicing sites of genes involved in channelopathies, we suggest the implementation of functional studies. To overcome the problem of the storage of samples to perform functional studies, we show two hypothetical ways: always keep at least one aliquot of the biological sample at −80 °C or implement in silico studies. Currently, in vitro tests are the most reliable and robust methods used to study non-coding variants. The main drawback is the storage space required for many samples in freezers at −80 °C. To get around the problem, we suggest freezing blood samples at −80 °C from the very time of collection until results are obtained from the DNA analysis. If the molecular and clinical analyses are inconclusive or incomplete, RNA could be extracted and an aliquot could be retained for future analysis. The preservation of extracts in tubes of max 2 mL is definitely a space-saving solution for long-term storage at −80 °C.

Where adequate storage of a sample is not possible, it would be useful to implement in silico studies, achieving a higher reliability, which allows for better classification of the non-coding variant found. In this regard, when using computational tools, it is necessary to periodically update the variant databases. All computational tools are based on or learn from the classification of validated variants; therefore, they can only be improved by acquiring more validated experimental data. In summary, in silico analysis can be performed to predict the significance of a non-coding variant, the analysis being based on available databases. At the same time, clinical variant databases should be updated with the results of validations. Advancements are essential for enhancing the accuracy of bioinformatic predictions and improving the assessment of the pathogenicity of variants, allowing the reclassification of variants of unknown significance in the future.

## Supplementary Materials

The following supporting information can be downloaded at: https://www.mdpi.com/article/10.3390/genes15101272/s1, Table S1: Types of LQTS and the respective causative mutated genes; Table S2: Types of BrS and the respective causative mutated genes; Table S3: Types of SQTS and the respective causative mutated genes; Table S4: Types of CPVT and the respective causative mutated genes; Table S5: This table shows the main genes involved in channelopathies and a comparison of the total number of variants with a pathological/likely pathological/VUS significance (in coding and non-coding sequences) with splice-site variants with the same clinical significances. Splice-site mutations are subclassified into pathogenic, likely pathogenic and VUSs to highlight the high number of VUSs with respect to the other established clinical significances (about 60%). The data were collected from the ClinVar website. P = pathogenic; LP = likely pathogenic; VUS = variant of unknown significance.
